# Lignocellulose Fractionation Using Recyclable Phosphoric Acid: Lignin, Cellulose, and Furfural Production

**DOI:** 10.1002/cssc.202002383

**Published:** 2020-12-10

**Authors:** Dennis Weidener, Walter Leitner, Pablo Domínguez de María, Holger Klose, Philipp M. Grande

**Affiliations:** ^1^ Institute of Bio- and Geosciences, Plant Sciences Forschungszentrum Jülich GmbH Wilhelm-Johnen-Straße 52428 Jülich Germany; ^2^ Institute of Technical and Macromolecular Chemistry (ITMC) RWTH Aachen University Worringer Weg 1 52074 Aachen Germany; ^3^ Institute for Biology I RWTH Aachen University Worringer Weg 3 52074 Aachen Germany; ^4^ Max-Planck-Institute for Chemical Energy Conversion Stiftstraße 34–36 45470 Mülheim an der Ruhr Germany; ^5^ Bioeconomy Science Center (BioSC), c/o Forschungszentrum Jülich Wilhelm-Johnen-Straße 52428 Jülich Germany; ^6^ Sustainable Momentum SL Av. Ansite 3, 4–6. 35011 Las Palmas de Gran Canaria Canary Islands Spain

**Keywords:** biomass conversion, fractionation, furfural, lignocellulose, phosphoric acid

## Abstract

The conversion of lignocellulose into its building blocks and their further transformation into valuable platform chemicals (e. g., furfural) are key technologies to move towards the use of renewable resources. This paper explored the disentanglement of lignocellulose into hemicellulose‐derived sugars, cellulose, and lignin in a biphasic solvent system (water/2‐methyltetrahydrofuran) using phosphoric acid as recyclable catalyst. Integrated with the biomass fractionation, in a second step hemicellulose‐derived sugars (mainly xylose) were converted to furfural, which was in situ extracted into 2‐methyltetrahydrofuran with high selectivity (70 %) and yield (56 wt %). To further increase the economic feasibility of the process, a downstream and recycling strategy enabled recovery of phosphoric acid without loss of process efficiency over four consecutive cycles. This outlines a more efficient and sustainable use of phosphoric acid as catalyst, as its inherent costs can be significantly lowered.

## Graphical abstract

**Lignin, cellulose, and furfural**: In a two‐step procedure, lignocellulose is first fractionated into lignin, cellulose, and monosaccharides to then synthesize furfural from the pentoses in the sugar hydrolyzate. Phosphoric acid is used as catalyst for the whole process and recycled over four cycles without loss in activity. The biphasic 2‐MTHF‐aqueous system allows for high selectivity, straightforward product separation, and downstream processing.

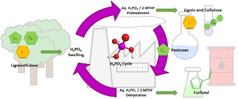

## Introduction

With fossil fuels depleting, the demand for alternative, more sustainable routes to produce fuels and chemicals is steadily growing. A reorientation of research towards renewable resources is putting biorefineries into the spotlight for the conversion and valorization of biomass‐derived streams.[^1^, ^2^, ^3^] Lignocellulose, the most abundant biomass on the planet,^[4]^ can be transformed into a large variety of platform chemicals, starting from lignin, cellulose, and hemicelluloses (e. g., producing xylose and other pentoses). Herein, furfural is a promising biogenic building block, chosen as one of the 30 most interesting value‐added chemicals from biomass by the US Department of Energy.^[5]^ It can be derived from xylose and other pentoses^[6]^ via dehydration and provides a platform for various different chemical transformations towards other molecules such as 2‐methyltetrahydrofuran (2‐MTHF), THF, levulinic acid, polyether building block, or cross linker in resins, among others.[^7^, ^8^, ^9^, ^10^, ^11^, ^12^] A biorefinery that could deliver on‐demand furfural with simultaneous utilization of lignin and cellulose may become a promising candidate for future practical implementation, as versatility and economics could be aligned.

The efficient acid‐catalyzed conversion of xylose or xylan to furfural has been broadly investigated. Biphasic or continuous processes have been proposed to reduce the degradation of furfural to humins, which is one of the main problems at the elevated temperatures needed for the reaction.[^13^, ^14^, ^15^] Likewise, the direct production of furfural from biomass has been reported and provides a rapid approach to produce it together with a cellulose‐enriched pulp, which can be used for further valorization.[^16^, ^17^, ^18^, ^19^] Different approaches have shown direct synthesis of furfural from lignocellulose with high selectivity, using, for example, mechanocatalytic strategies,^[20]^ heterogeneous catalysts,^[21]^ or homogeneous catalysts.^[22]^ More holistic lignocellulose fractionation concepts like organosolv^[23]^ are designed to valorize all of the main lignocellulose components. The recently developed OrganoCat pretreatment^[24*–*26]^ uses an acid catalyst [biogenic; oxalic acid or 2,5‐furandicarboxylic acid, (FDCA)] and a biphasic solvent system, extracting lignin in situ into the second 2‐MTHF phase to reduce further degradation of lignin in the acidic solution and make product separation more straightforward. Herein, the downstream processing of hemicellulose‐derived monosaccharides (mainly pentoses) remains challenging. A promising option may be to convert pentoses into furfural, using the aqueous effluent from the pretreatment directly. Thus, the acid catalyst can have a double function: fractionating lignocellulose and subsequently dehydrating pentoses. For that concept, a biphasic system would facilitate the in situ extraction of the formed furfural.^[22]^ Typically, phosphoric acid is known to be a cellulose solvent (when concentrated, to produce swollen cellulose) and an efficient acidic catalyst for polysaccharide hydrolysis.[^27^, ^28^] Several process concepts have been developed using phosphoric acid, for example, in combination with mechanical treatment,^[29]^ or by adding organic solvents like acetone or ethanol.[^30^, ^31^, ^32^, ^33^, ^34^] The practical use of phosphoric acid as catalyst may be hampered by its costs;^[35]^ however, such biorefinery concepts might become economically viable with optimal heat integration, efficient recycling of the acid, and lowering the energy consumption of the process. Thus, integrated concepts, in which several marketable products are generated and the catalyst can be recycled, are needed. In this work, a holistic concept is put forth, evaluating the use of phosphoric acid for lignocellulose pretreatment and pentose dehydration in a biphasic system. By using the same catalyst‐solvent system for both reaction steps, downstream processing might be simplified, although the use of other biogenic solvents [e. g., cyclopentyl methyl ether (CPME)[^36^, ^37^, ^38^]] may be considered for the furfural production as well. The process will deliver lignin, cellulose, and furfural as main outcome streams. Importantly, the integrated concept enables the straightforward recycling of phosphoric acid, water, and solvent, thus adding promising features for its future practical implementation.

## Results and Discussion

**Figure 1:**
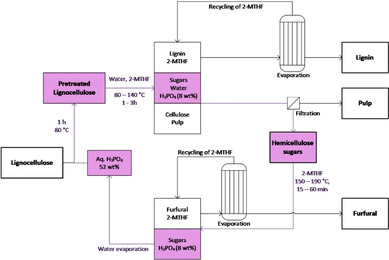
Biomass fractionation and integrated furfural production using phosphoric acid as catalyst. The process consists of a swelling step of dried lignocellulosic biomass at 80 °C, followed by a biphasic fractionation step, using water and 2‐MTHF as solvents. After phase separation, the sugar containing hydrolyzate is used for another biphasic reaction step to produce and extract furfural. After phase separation and water evaporation, the phosphoric acid can be reused.

The overall concept of the proposed lignocellulose pretreatment, fractionation, and conversion is depicted in Figure 1. In brief, lignocellulose processing starts with a swelling step to increase the accessibility of the cellulose‐enriched pulp. To that end, the lignocellulose is mixed with phosphoric acid (52 wt %) and heated to 80 °C for 1 h. Subsequently, hemicelluloses, lignin, and cellulose are fractionated with an OrganoCat‐like approach, using the same phosphoric acid, and recycling the water and the solvent to increase the economic and sustainability potential of the process.

The swelling of the biomass (1000 g L^−1^) in phosphoric acid (52 wt % in water) is performed at 80 °C to avoid formation of humins. In the fractionation step, the acid is diluted with water to 8 wt %. 2‐MTHF (equal volume to the aqueous phase) is added as a second phase, resulting in a biomass loading of 100 g L^−1^ referring to the aqueous phase. With that amount of phosphoric acid a pH of approximately 1 is achieved for the fractionation, which has shown to provide an efficient (and selective) hydrolysis of the hemicelluloses sugars.[^24^, ^26^] A lower pH would improve the hydrolysis but would also increase the degradation of sugars and lignin during the fractionation step. After removing the organic phase and the solid pulp, new 2‐MTHF (equal volume to the aqueous phase) is added to the hydrolysate, and the resulting pentoses are converted to furfural at 150–190 °C with reaction times of up to 1 h. Higher temperatures and longer reaction times lead to the uncontrolled formation of furfural and other undesired degradation products.[^24^, ^25^, ^26^] After phase separation, furfural is recovered from the organic phase and the aqueous phase, containing remaining sugars (79 % xylose, see the Supporting Information, Table S6) and phosphoric acid is concentrated to be reused in the lignocellulose swelling step.

**Figure 2:**
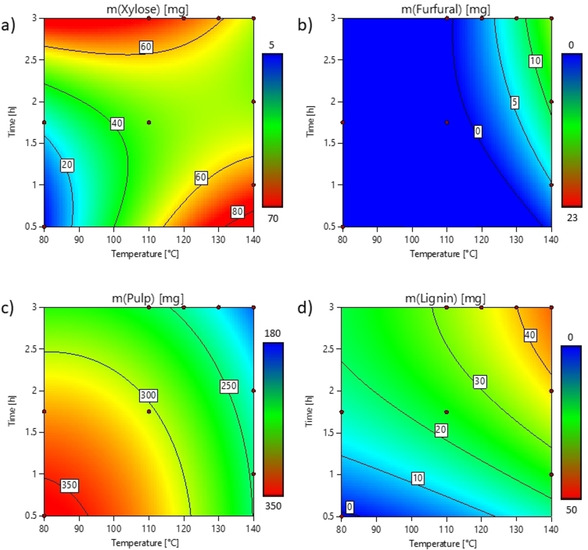
Results of DOE study for the lignocellulose fractionation with a swelling pre‐step (1 h, 80 °C) using Design‐Expert® Software Version 12.^[40]^ (a) Amount of xylose; (b) amount of furfural; (c) amount of cellulose‐enriched pulp; and (d) amount of lignin for different temperatures and reaction times. Values are given by the contour lines and increase from blue to red. All data is displayed in the Supporting Information, Table S1.

To validate the described process, 10 mm beech wood chips were used as a prototypical substrate, which contain more than 85 % xylan^[39]^ in the hemicelluloses and provide a defined composition of hemicelluloses, cellulose, and lignin. The reactions were performed in a 20 mL stainless‐steel high‐pressure reactor. Statistical design of experiments (DOE) was used to identify optimal conditions for the different reaction steps. Temperatures between 80–140 °C and reaction times between 1–3 h were evaluated for the fractionation step (Figure 2).

The results of the fractionation condition screening are shown in Figure 2. At longer times and higher temperatures, the amount of extracted lignin increases, and the amount of cellulose‐enriched pulp is reduced. The amount of xylose diminishes at harsher conditions, as it starts to be converted into furfural. Depending on the desired products the fractionation can be tuned accordingly to prioritize the needs. For a holistic biorefinery, a fine‐tuning depending on the biomass recalcitrance and product requirements can be made in terms of reaction time and temperature. Therefore, the focus can be shifted, for example, from maximal xylose/furfural selectivity and yield towards a balance between furfural yield and amount of extracted lignin.

**Figure 3:**
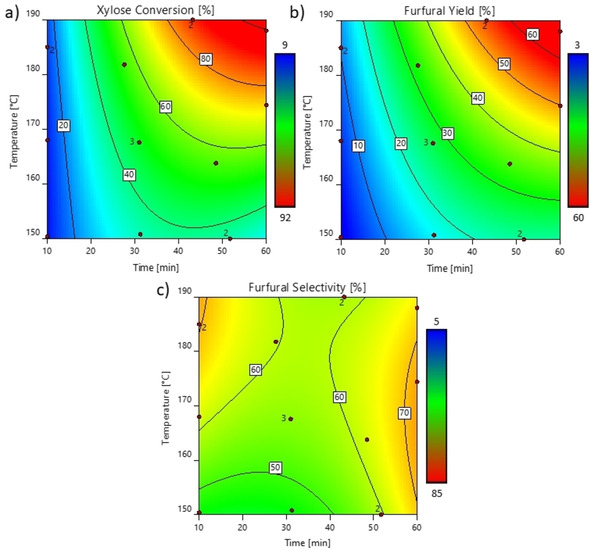
Results of DOE study for the subsequent furfural production from hydrolysate obtained from the lignocellulose swelling and fractionation (swelling: 1 h, 80 °C; fractionation: 3 h, 140 °C) using Design‐Expert® Software Version 12.^[40]^ The numbers indicate the repetitions of experiments. (a) Xylose conversion, based on the initial amount of xylose; (b) furfural yield, based on initial amount of xylose; and (c) selectivity, for different temperatures and reaction times. Values are given by the contour lines and increase from blue to red. All data is displayed in the Supporting Information, Table S2.

To produce furfural with high selectivity and yield, the screening (Figure 3) showed an optimum at a temperature around 175 °C and a reaction time of approximately 1 h, where the selectivity is >70 %, and yield is >56 %. These results are in a comparable range as results obtained by other furfural production systems from lignocellulose (e. g., from beech wood by Carrasquillo‐Flores et al.: furfural selectivity 74 %^[20]^). Although higher temperatures improve the xylose conversion, the selectivity for furfural is lower. Degradation products like humins are challenging to valorize. Therefore, incomplete conversion with high selectivity is favorable over high degradation, which can be compensated by multiple cycles reusing the residual xylose. To optimize the furfural synthesis, a compromise between selectivity and degradation, energy consumption, heat integration, and others needs to be reached. The hydrolysate of the biphasic fractionation step was used at identical conditions with and without 2‐MTHF as a second liquid phase. The in situ extraction of furfural into 2‐MTHF at low xylose conversion (9 %; at 168 °C, 10 min) led to a selectivity of >80 % whereas up to 70 % furfural selectivity was achieved at high xylose conversion (80 %; at 174 °C, 60 min). In the monophasic system only 71 % selectivity (6 % xylose conversion) and 33 % (88 % xylose conversion), respectively, was achieved (see the Supporting Information, Table S2). Thus, the biphasic system increases the overall selectivity of the process at high conversion rates, which is consistent with the literature.[^15^, ^41^] It enables a straightforward downstream processing, as furfural can be isolated by evaporation of 2‐MTHF (or subsequently be directly valorized in the organic effluent).

**Figure 4:**
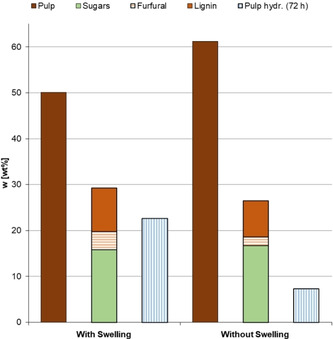
Comparison of representative fractionation conditions with and without a preceding swelling: Cellulose‐enriched pulp and the liquid fractions (sugars, furfural formation during fractionation, and lignin), as well as glucose after 72 h of pulp hydrolysis (given as wt % of glucose per hydrolyzed pulp). All data is displayed in the Supporting Information, Table S1.

As depicted in Figure 4, the swelling step promotes the efficiency of the fractionation in multiple ways. Less cellulose‐enriched pulp and higher amounts of extractives were derived from beech wood material processed with the swelling step (Figure 4; beige, green, orange, and brown bars). When digested with Accellerase® 1500 for 72 h, the pulps exhibited almost 3‐fold higher glucose release rates (Figure 4, blue bars). This is presumably caused by an amorphization of the cellulose,[^42^, ^43^] enabling even higher accessibility of the material for the diluted acid and subsequently the enzymatic hydrolysis.^[44]^ The increased delignification improves the hydrolysis as well,^[45]^ while generating more lignin for valorization. Higher pulp accessibility can be beneficial for subsequent fermentation‐based strategies (e. g., ethanol or alkenes),^[46]^ or chemical conversions to 5‐hydroxymethylfurufural and/or levulinic acid, two relevant chemical building blocks.[^47^, ^48^]

**Figure 5:**
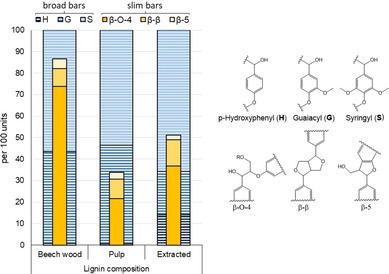
Composition of lignin in beech wood, pulp, and the organic phase of the fractionation. The ratios of the monomer units H, G, and S are displayed in broad bars, and the amount of oxygen containing linkages β‐O‐4, β‐β, and β‐5 is displayed in slim bars. All data is displayed in the Supporting Information, Table S4; a representative HSQC‐NMR spectrum is displayed in Figure S1.

Another important aspect of the proposed biorefinery concept is the extraction and recovery of lignin. The valorization of lignin for macromolecular applications or as a feedstock for chemicals can boost the revenues of a biorefinery.[^49^, ^50^] Different strategies focus on lignin depolymerization by cleaving the oxygen‐rich linkages between the monomers [*p*‐hydroxyphenyl (H), guaiacyl (G), and syringyl (S)], such as β‐O‐4, β‐β, and β‐5.[^51^, ^52^, ^53^] Therefore, it is important to keep these bonds largely intact and prevent re‐condensation during the fractionation and lignin extraction. To evaluate the effect of the conditions applied here, the ratio between H‐, G‐, and S‐units and the amount of linkages between the units was determined for raw beech wood, residual lignin in the cellulose‐enriched pulp, and lignin in the organic extract. Extracted lignin was dried to determine the dry mass and directly dissolved in DMSO‐d_6_, whereas the raw material and pulp where observed using a technique described by Cheng et al.^[54]^ The H/G/S ratio (Figure 5, broad bars) is slightly altered, showing an increased proportion of H‐units in the extracted lignin. The amount of the most abundant linkage (β‐O‐4, Figure 5, slim orange bars) is reduced to approximately half of the amount found in untreated beech wood, a less severe reduction when compared to similar fractionation systems.^[26]^ A higher amount of cleavable linkages in the extracted lignin compared to, for example, Kraft lignin can be favorable for better lignin depolymerization.

**Figure 6:**
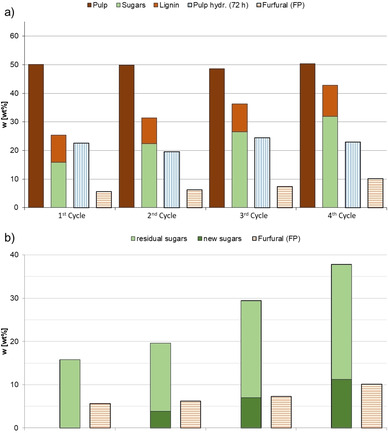
Recycling of phosphoric acid over four complete cycles of lignocellulosic biomass swelling (1 h, 80 °C), fractionation (3 h, 140 °C), and furfural synthesis (1 h, 174 °C). (a) Yields of the fractions cellulose‐enriched pulp and lignin (given as wt % relative to initial biomass) after fractionation step, glucose release from subsequent pulp hydrolysis using Accellerase® 1500 (given as wt % of glucose per hydrolyzed pulp after 72 h). (b) Sugar concentrations of residual sugar from the previous cycle(s) (dark green bars), newly extracted sugars from the biomass (light green bars), and furfural yield (brown bars) from subsequent furfural synthesis step using the sugar hydrolysate of the fractionation (given as wt % of sugars and furfural relative to initial biomass). All data is displayed in the Supporting Information, Table S3.

To improve the economic and environmental feasibility of the process, recycling experiments were performed, following the single‐step experimental protocols. After successful fractionation of the biomass and separation of the liquid phases and the pulp, the resulting hydrolysate was used to produce furfural, closing the loop as shown in Figure 1. Afterwards, the aqueous phase of the furfural production was concentrated under reduced pressure and used again for the next cycle of swelling and fractionation. This procedure was repeated during four consecutive cycles to assess the resulting extractives and the overall performance. Remarkably, the efficiency of the fractionation (extracted lignin and residual pulp yields) is constant with each run, as well as the hydrolysis of the cellulose enriched pulp (Figure 6a). The amount of reducing sugars in the hydrolysate accumulates during the four cycles (Figure 6b), due to recycling the aqueous phase from the furfural production, which does not provide full conversion of sugars. Consequently, the amount of produced furfural increases as well. Overall, the excellent recyclability depicts a promising holistic approach that showed a stable performance of the catalyst and solvent during the four cycles. This is of importance to reduce material costs in particular for 2‐MTHF and phosphoric acid in order to reach sustainability and economic figures.

## Conclusions

A novel integrated process for lignocellulose pretreatment and fractionation, coupled with the conversion of hemicelluloses sugars into furfural, maintaining the same solvent/catalyst system has been described. A preceding swelling step increases overall efficiency of the fractionation, yielding high‐quality lignin and cellulosic pulp, which exhibits higher enzymatic hydrolysis rates than without pre‐swelling. Furfural was produced from the resulting hydrolysate of the fractionation with yields of up to 57 wt % (5.7 g L^−1^ h^−1^ within the first hour) at high selectivity of up to 70 % and was straightforwardly separated by in situ extraction from the aqueous phase using 2‐methyltetrahydrofuran as secondary phase. Phosphoric acid was reused for four consecutive cycles of the process without decreasing the process efficiency. Both biphasic reactions enable the straightforward product separation without the addition of new chemicals in the second step and provide low degradation of both lignin and furfural during the respective reactions. The obtained lignin kept half of the β‐O‐4 linkage content, which can be of advantage for further applications. The presented process envisages a holistic valorization of the lignocellulosic biomass, with a high potential towards utility and economics of lignocellulosic biorefineries.

## Experimental Section

**Materials**: Phosphoric acid (85 wt %) and 2‐MTHF were obtained from Carl Roth, and Avicel PH 101 was obtained from Sigma‐Aldrich and used without further purification. Accellerase® 1500 was kindly donated by Genencor (The Netherlands). Beech wood was obtained from local suppliers (the particle size was generated by a cutting mill with a 10 mm sieve) and dried at 50 °C until constant weight (≈24 h).

**Procedure for lignocellulose fractionation**: In a 20 mL high‐pressure reactor, 400 mg beech wood lignocellulose, 200 μL ultrapure water, and 200 μL phosphoric acid (85 wt %) were mixed and heated at 80 °C for 1 h. Afterwards, 3.6 mL ultrapure water and 4 mL 2‐MTHF were added. The stainless‐steel high‐pressure reactor was closed, and 10 bar of argon was added to minimize 2‐MTHF evaporation. The reactor was heated to 140 °C, and the mixture was stirred for 3 h. After cooling the reactor down to room temperature, the liquid phases were separated by decantation, and the cellulose‐enriched pulp was filtered. The sugar concentrations were determined using HPLC and PAHBAH (explanation of analytical methods see below). The solid residue was washed with distilled water until neutral pH and dried until constant weight. Lignin was obtained by evaporation of 2‐MTHF and analyzed using NMR spectroscopy. All experiments were conducted as single experiments or with replicates as indicated in the text.

**Procedure for furfural production**: In a 20 mL high‐pressure reactor, 2 mL of hydrolysate from the lignocellulose fractionation and 2 mL 2‐MTHF were added. The reactor was closed and pressurized with 10 bar of argon to minimize 2‐MTHF evaporation. The reactor was heated to 190 °C and stirred for 20 min. After cooling the reactor down to room temperature, the liquid phases where separated by decantation. The sugar concentrations were determined in the aqueous phase via HPLC and PAHBAH (explanation of analytical methods see below). Furfural was quantified in the organic phase via GC. All experiments were conducted as single experiments or with replicates as indicated in the text.

**Lignin NMR spectroscopy**: NMR measurements where conducted on a Bruker AS400 (400 MHz) Spectrometer. Approximately 50 mg of lignin was dissolved in 0.5 mL of DMSO‐d_6_. ^1^H‐^13^C heteronuclear single quantum coherence (HSQC) (measurement time 7 : 20 h) measurements were used to determine the type of linkages present in the respective lignin fractions of the reactions. The corresponding signals of each substructure were integrated and quantified using mesitylene as internal standard.

**HPLC monosaccharide analysis**: HPLC analysis was conducted on a Jasco HPLC equipped with a SUGARSH1011 column with a 0.01 wt % aqueous acetic acid solution as eluent. The flow rate was set to 0.6 mL min^−1^, and undiluted samples of 30 μL were injected. Amounts of xylose and glucose present in the hemicelluloses fraction were determined based on calibration curves, using commercially available authentic substrates.

**PAHBAH quantification of reducing sugars**: The concentration of reducing sugars in the aqueous hydrolysate was determined using the PAHBAH (4‐hydroxybenzoic acid hydrazide) method as described by Lever.^[55]^ The hydrolysate was diluted by a factor of 100. The resulting reaction mixture was diluted by a factor of 2 and measured on a BioTek Power Wave HT UV/Vis spectrometer at 410 nm.

**Enzymatic hydrolysis**: Hydrolysis of different cellulose‐rich substrates was carried out in an Eppendorf Thermomixer Comfort using 1.5 mL Eppendorf vials. For each reaction, 20 mg of the substrate and 10 μL Accellerase® 1500 (60 FPU mL^−1^ and 82 CBU mL^−1^, Genencor, The Netherlands) were dissolved in 1 mL citrate buffer (pH=4.5) and shaken at 50 °C for a specific time. Afterwards, the samples were heated to 100 °C for 5 min. The glucose concentration was determined using a Glucose (HK) Assay Kit obtained from Sigma‐Aldrich and a BioTek Power Wave HT UV/Vis spectrometer.

**Furfural quantification (GC)**: GC measurements to quantify the amount of furfural in the 2‐MTHF phase were conducted using a 30 m HP‐Innowax column, with helium as carrier gas with a flow rate of 1.5 mL min^−1^ and a flame‐ionization detector. The initial temperature was 50 °C, raised by 8 °C min^−1^ to 250 °C and left at 250 °C for 5 min. Quantification was done using *n*‐decane as the internal standard.

**Furfural quantification (^1^H NMR spectroscopy)**: NMR measurements where conducted on a Bruker AS400 (400 MHz) Spectrometer. Approximately 35 μL of the aqueous phase or the 2‐MTHF phase was dissolved in 0.5 mL of DMSO‐d_6_. ^1^H NMR measurements were used to determine the amount of furfural using mesitylene as internal standard.

**Design of Experiments**: The reaction parameters for the lignocellulose fractionation and the furfural production were chosen by using DOE with Design‐Expert® Software Version 12^[40]^ in order to generate a response surface for each response factor, using a quadratic model. Equations (1)–(3) were used:

(1) $$\mathrm{Conversion}:\;X_{i}=\frac{n_{substrate}\left(t=0\right)-n_{substrate}\left(t\right)}{n_{substrate}\left(t=0\right)}$$

(2) $$\mathrm{Yield}:\;Y_{i}=\frac{n_{product}\left(t\right)}{n_{substrate}\left(t=0\right)}$$

(3) $$\mathrm{Selectivity}:\;S_{i}=\frac{Y_{i}}{X_{i}}$$

**Phosphoric acid distribution**: In a single experiment, 200 μL phosphoric acid (85 wt %), 3.8 mL water, and 4 mL 2‐MTHF were mixed, heated to 140 °C, and stirred for 3 h. After cooling to room temperature, the phases were separated via decantation. The 2‐MTHF phase was measured using ^31^P NMR spectroscopy, and the amount of phosphoric acid was determined gravimetrically after evaporation of 2‐MTHF. The phosphoric acid concentration in the aqueous phase was determined before and after heating to 140 °C via titration with aqueous sodium hydroxide.

## Conflict of interest

The authors declare no conflict of interest.

## Supporting information

As a service to our authors and readers, this journal provides supporting information supplied by the authors. Such materials are peer reviewed and may be re‐organized for online delivery, but are not copy‐edited or typeset. Technical support issues arising from supporting information (other than missing files) should be addressed to the authors.

Supplementary
