# Supplementary material for: Lignocellulose Fractionation Using Recyclable Phosphoric Acid: Lignin, Cellulose, and Furfural Production
Source: ChemSusChem. 2020 Dec 10;14(3):909–16. doi: 10.1002/cssc.202002383 (PMC7898823; doi:10.1002/cssc.202002383)
Supplement: Supplementary file 1 — Supplementary [file CSSC-14-909-s001.pdf]

ChemSusChem

Supporting Information

Lignocellulose Fractionation Using Recyclable Phosphoric Acid: Lignin, Cellulose, and Furfural Production

Dennis Weidener, Walter Leitner, Pablo Domínguez de María, Holger Klose, and Philipp M. Grande*© 2020 The Authors. ChemSusChem published by Wiley-VCH GmbH. This is an open access article under the terms of the Creative Commons Attribution License, which permits use, distribution and reproduction in any medium, provided the original work is properly cited.

Author Contributions

D.W. Writing – original draft:Supporting
W.L. Supervision:Supporting
P.D. Supervision:Supporting
H.K. Supervision:Supporting

Table S1: Results of fractionation step at different temperatures and times. Raw biomass was swollen at 80 °C for 1 h with 0.4 mL of phosphoric acid (~52 wt%). After dilution with water and addition of 2-MTHF, reaction times and temperatures were applied as indicated.

	Temperature [°C]	Time [h]	Pulp [wt%]	Lignin [wt%]	Xylose [wt%]
	140	1.00	63.7	5.3	18.3
	140	2.00	55.9	7.0	18.0
	140	3.00	50.1	9.6	15.8
	110	3.00	65.0	7.5	16.2
	120	3.00	59.6	9.0	17.3
	130	3.00	53.9	12.1	17.2
	70	1.75	82.5	2.5	4.1
	80	1.75	81.1	4.8	6.5
	150	3.00	45.6	3.8	8.2
	80	0.50	88.6	0.0	1.3
	110	1.75	78.7	6.4	7.8
	150	1.75	49.5	15.0	10.8
*	140	3.00	61.2	7.8	16.7

* without preceding swelling

Table S2: Screening of furfural production step at different times and temperatures. A hydrolysate was used that was produced using swelling for 1 h at 80 °C in phosphoric acid (52 wt%) and fractionation after dilution with water and addition of an equal amount of 2-MTHF at 140 °C for 3 h, achieving a xylose concentration of 15.7 g L^{-1}

	Temperature [°C]	Time [min]	Furfural [%]	Xylose [g/L]	Conversion [%]	Selectivity [%]
	168	10	7.4	14.3	8.8	84.6
	185	10	7.3	13.4	14.4	50.4
	164	49	40.4	7.0	55.6	72.7
	182	28	47.5	5.0	68.1	69.8
	150	52	21.8	9.8	37.9	57.4
	190	43	58.7	1.9	88.0	66.7
	150	10	6.4	13.1	16.7	38.3
	190	43	55.2	1.4	90.8	60.8
	168	31	3.0	8.5	45.9	6.5
	150	52	20.9	10.3	34.2	61.0
	188	60	56.2	1.3	91.6	61.3
	168	31	31.6	8.3	47.4	66.8
	185	10	7.7	14.2	9.9	77.5
	151	31	15.1	11.0	29.7	51.0
	168	31	26.6	9.2	41.3	64.4
	174	60	57.2	3.1	80.4	71.1
	190	20	47.1	4.0	74.6	63.2
*	168	10	7.4	14.3	8.8	84.6
*	174	60	7.3	13.4	14.4	50.4

* monophasic system

Table S3: Fractions from four consecutive cycles of lignocellulose processing using phosphoric acid.

Fraction:	1st Cycle	2nd Cycle	3rd Cycle	4th Cycle
Pulp [wt%]	50.1	49.9	48.6	50.4
Sugars [wt%]	15.8	22.4	26.5	31.9
Lignin [wt%]	9.6	9.1	9.7	11.0
Pulp hydr. (72 h) [wt%]	22.6	19.5	24.4	23.0
Furfural (second reaction step) [wt%]	5.5	6.1	7.2	10.0

Table S4: Composition of lignin in raw biomass, pulp and extracted with phosphoric acid pretreatment, as well as extracted using OrganoCat with oxalic acid (OC) determined via ^{1}H-^{13}C-HSQC-NMR.

	Lignin composition			
	Beech wood	Pulp	Extracted	OC
H [%]	0.7	0.5	14.2	8.7
G [%]	42.9	46.0	20.0	36.2
S [%]	56.4	53.5	65.8	55.1
β-O-4 [%]	73.7	21.6	36.7	25.2
β-β [%]	8.3	9.0	12.3	9.4
β-5 [%]	4.5	3.1	2.1	1.6

Table S5: Analysis of furfural distribution at different temperatures. A hydrolysate (15 g L^{-1}) from the first reaction step was used. 4 mL of hydrolysate and 4 mL of 2-MTHF were used. Furfural in water and 2-MTHF was determined using ^{1}H NMR spectroscopy.

Temperature [°C]	Time [min]	Furfural (2-MTHF) [mg]	Furfural ($H_2 0$) [mg]	Furfural (2-MTHF) [%]	Furfural ($H_2 0$) [%]
150	25	2.0	0.5	79.1	20.9
190	25	19.5	4.6	80.9	19.1

Table S6: Monosaccharide composition of beech wood hemicellulose.

Fucose [%]	Rhamnose [%]	Arabinose [%]	Galactose [%]	Glucose [%]	Xylose [%]	Mannose [%]	Galacturonic Acid [%]	Glucuronic Acid [%]
0.0	1.5	1.6	2.0	8.8	78.6	4.4	3.3	0.0

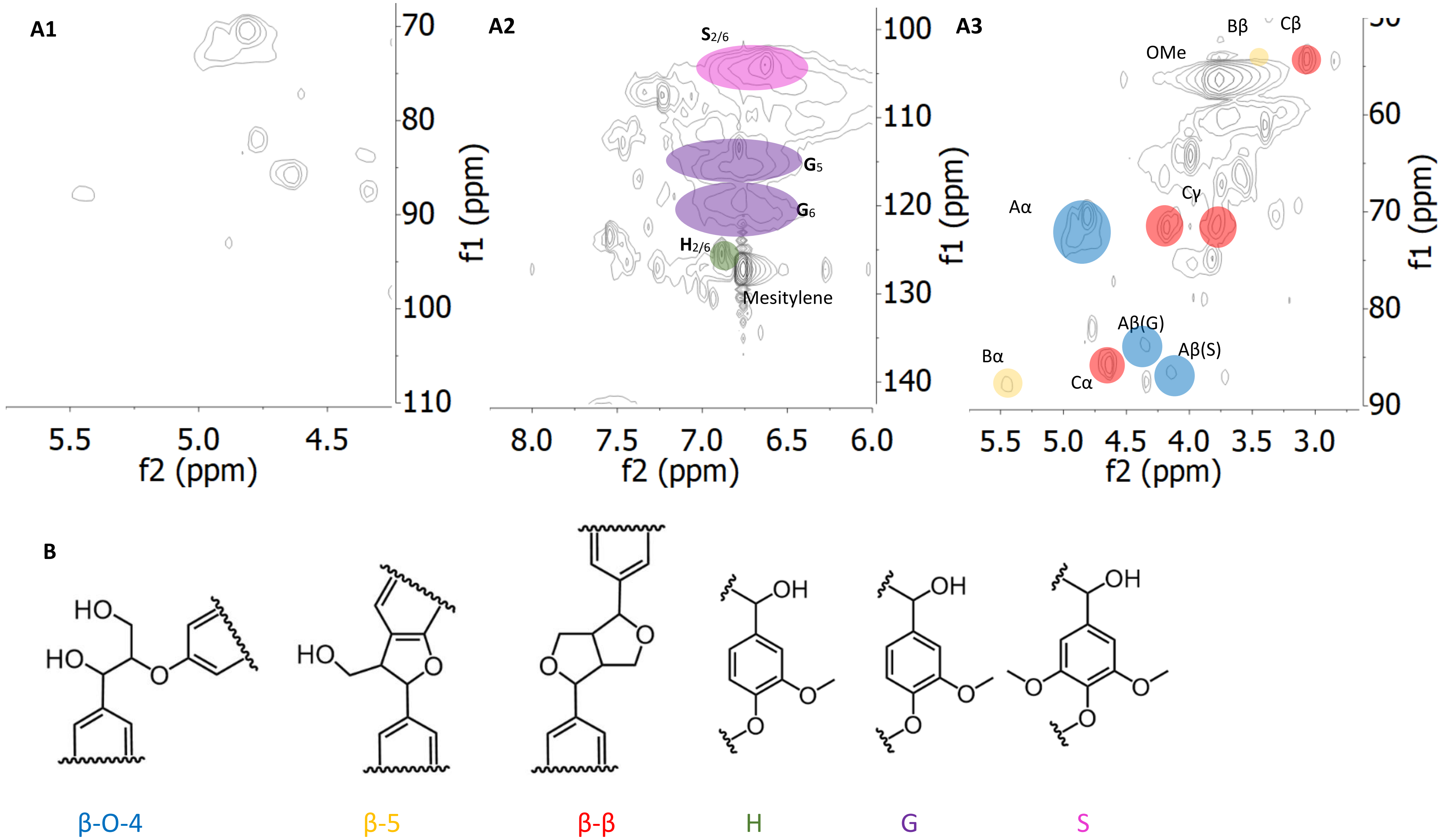

Figure S1: Expansion of ^{1}H-^{13}C-HSQC spectrum of lignin extracted from beech wood using phosphoric acid. A1 (Carbohydrate region), A2 (aromatic region), A3 (aliphatic region) and B (investigated linkages and monomer units).
